# Advancements and Innovation Trends of Information Technology Empowering Elderly Care Community Services Based on CiteSpace and VOSViewer

**DOI:** 10.3390/healthcare13131628

**Published:** 2025-07-07

**Authors:** Yanxiu Wang, Zichun Shao, Zhen Tian, Junming Chen

**Affiliations:** 1 Faculty of Humanities and Arts, Macau University of Science and Technology, Macao 999078, China; 2240020192@student.must.edu.mo (Y.W.); 220015871@student.must.edu.mo (Z.S.); 2 James Watt School of Engineering, University of Glasgow, Glasgow G12 8QQ, UK; 2620920Z@student.gla.ac.uk

**Keywords:** information technology, computer systems, health services for the aged, bibliometrics

## Abstract

**Background**: In elderly community services, information technology is reshaping the daily lives of older adults in unprecedented ways. It effectively addresses the issue of frailty in the community by strengthening support networks and dynamic risk management. Despite its vast potential, there remains a need to explore further enabling methods in the realm of elderly community services. **Objectives**: This study aims to provide a significant theoretical and practical foundation for information technology in this field by systematically analyzing the progress and trends of digital transformation facilitated by information technology. Materials and method: To map the advancements and emerging trends in this evolving field, this study conducts a bibliometric analysis of 461 relevant publications from the Web of Science Core Collection (2004–2024). The research employs bibliometric methods and utilizes tools such as CiteSpace and VOSViewer to analyze collaborations, keywords, and citations, as well as to perform data visualization. **Results**: The findings indicate that current research hotspots mainly focus on “community care”, “access to care”, “technology”, and “older adults”.Potential development trends include (1) further exploration of information technology in elderly care to provide more precise health management solutions; (2) systematically building community elderly service systems to offer more detailed elderly care services; (3) strengthening interdisciplinary information sharing and research collaboration to drive innovation in community elderly care models; and (4) introducing targeted policy and financial support to improve the specific implementation framework of information technology in elderly community services. **Conclusions**: This study provides empirical support for the development of relevant theories and practices. Furthermore, the research outcomes offer valuable insights into business opportunities for practitioners and provide important recommendations for formulating elderly service policies.

## 1. Introduction

### 1.1. Research Background and Motivation

The global aging population presents significant challenges for community care systems, with digital transformation, particularly in digital health, offering crucial solutions. As an integral part of this transformation and social digitalization, information technology-enabled community services for older adults can address social vulnerability by strengthening support networks and enabling dynamic risk management, thereby helping prevent physical and cognitive decline [1,2,3]. As technology advances, an increasing number of individuals realize the value and potential applications of information technology (IT) in empowering community elderly services. In the field of elderly care community services, applications of IT, such as telemedicine systems [4,5,6,7,8], intelligent health monitoring devices [5,9,10], and communication platforms [11,12,13], demonstrate significant advantages in improving service accessibility, reducing costs, enhancing self-management of health, and fostering social engagement. These contribute to increased efficiency, quality, and the ability to meet the diverse needs of older adults. A research report indicates that the market size of elderly services is expected to increase from USD 1044.86 billion to USD 1132.54 billion between 2024 and 2025, reflecting a compound annual growth rate (CAGR) of 8.4% [14]. Given the massive demand for elderly services in the rapidly growing market, it is essential to examine the empowerment of elderly community services through IT and its applications.

Research on IT-empowered elderly community services began in the early 21st century with basic systems that had limitations in functionality and user experience [15,16,17]. The rapid development of the internet and mobile technologies has led to the emergence of more sophisticated applications, including telemedicine [7,8,18], electronic health records [19,20], and mental health intervention platforms [21]. Recently, the integration of artificial intelligence (AI) and Internet of Things (IoT) technologies has opened new avenues for precise health management and life care (e.g., virtual care [4], intelligent health monitoring [10]), but simultaneously raises greater demands on provider technical competencies and elderly digital literacy, alongside persistent challenges in data security and privacy.

Information-based community services can effectively mitigate the challenges posed by an aging population and enhance the quality of life and well-being of older persons [22]. A thorough understanding of the application status and existing IT issues in elderly community services can be attained through systematic and visual research, informing future research and practice [23]. Therefore, it is necessary to conduct a systematic review of the application of information technology in elderly care community services to fully understand the current research status, key trends, and technical bottlenecks in the field, summarize development trends and challenges, and guide future research.

### 1.2. Research Gap

Currently, research on information-empowered elderly community services encompasses various domains, primarily including telemedicine [24], intelligent health monitoring [25], and community support [26]. Research on telemedicine focuses on providing convenient medical services through video conferencing and mobile health applications to reduce hospital visits by older persons and enhance the accessibility of medical services [7,24]. The telemedicine model significantly improves their health management and quality of life, especially for older adults living in remote areas or those with mobility issues. Investigations into intelligent health monitoring emphasize using wearable devices and sensors to track the health status of elderly individuals in real-time, encompassing metrics such as heart rate, blood pressure, and activity levels [25,27]. This monitoring approach facilitates the timely detection of potential health issues and offers personalized health management advice to reduce disease risks and healthcare expenses [27]. Furthermore, research on community support systems focuses on the development and optimization of information platforms to effectively connect older persons with community resources, offering life support and opportunities for social engagement [10,20,26].

A systematic analysis of relevant research in elderly care services is crucial for understanding and optimizing existing service models. Presently, the empowerment of IT in elderly care services has emerged as a research hotspot, with a growing amount of related review literature. Dai Baozhen et al. [28] reviewed the existing research status of elderly health security in rural China, analyzing 31 related articles and primarily addressing the influence of policy, economic, and social factors on elderly health security. For instance, Xie Yuchen et al. [29] reviewed community-based elderly care services from the consumer perspective, examining service accessibility and satisfaction. Subsequently, Nancy Matthew-Maich et al. [30] reviewed the application of mobile health technology in chronic disease management for older adults, analyzing around 42 articles to assess the effectiveness and user acceptance of the technology.

However, these review articles primarily analyzed the literature through the subjective evaluation of several samples. Conversely, bibliometric analysis is a statistical and quantitative approach that can use extensive objective data for analysis, thereby reviewing specific research domains and reducing the impact of subjectivity and bias. Furthermore, quantitatively, bibliometric analysis can reveal research hotspots, trends, and development patterns, offering significant references for scientific evaluation and academic research [31]. Among them, CiteSpace [32] and VOSViewer [33] facilitate researchers in identifying research hotspots, constructing knowledge maps, and elucidating research trends via visualization and network analysis of literature data [34]. Therefore, this paper uses CiteSpace and VOSViewer software to perform a comprehensive and quantitative analysis of literature related to information-empowered elderly community services, aiming to explore research dynamics, key issues, and development trends in this domain.

### 1.3. Research Goals and Significance

This study aims to systematically examine 461 pieces of literature from 2004 to 2024, elucidating advancements and trends in IT to facilitate elder care community services, identifying key participants and research hotspots, and establishing a knowledge framework in this domain. The specific research objectives are as follows:

(1) To identify key individuals, institutions, and nations contributing to this field.
(2) To develop a comprehensive research framework.
(3) To suggest future research avenues and enhancement strategies.

This study establishes a crucial foundation for the theory and practice of IT in facilitating elder care community services. Moreover, this study is advantageous for stimulating the investigation of business opportunities, serving as a reference for policy development, fostering interdisciplinary research, and facilitating advancements in technology and applications. This research promotes social cohesion and sustainable development, enhances the quality of life for the elderly, and possesses substantial academic merit and practical societal relevance.

## 2. Research Methods

**Figure 1:**
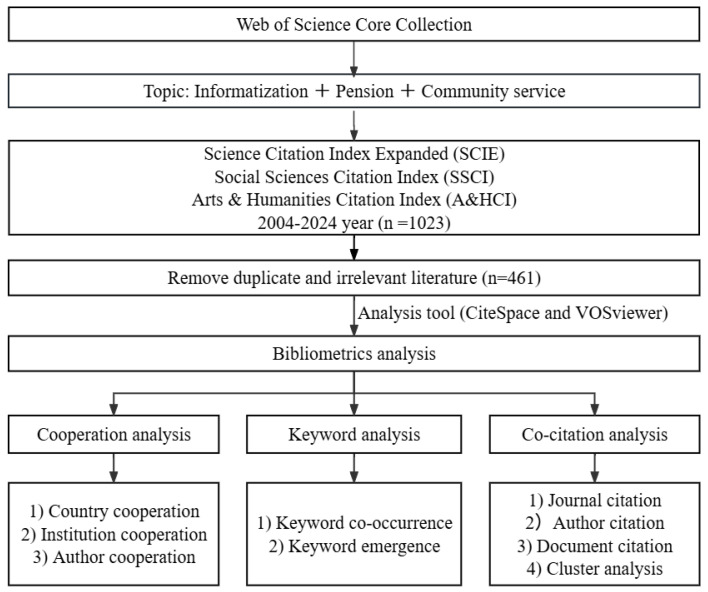
Research framework.

Bibliometrics is a method that elucidates the structure, dynamics, and patterns of scientific research via the quantitative analysis of literature data, primarily including co-word analysis and co-citation analysis of the literature [35]. Based on bibliometrics, this study reviews the literature from three dimensions: cooperation relationships, keywords, and co-cited references, to enhance a comprehensive understanding of the field of IT-enabled elder care services. Figure 1 illustrates the framework of the article. The analysis of cooperative relationships is performed from a macro-to-micro perspective, encompassing national cooperation, institutional cooperation, and author cooperation. Keyword analysis primarily includes co-occurrence and burst analysis of keywords. Co-citation analysis encompasses journal citation analysis, author citation analysis, document citation analysis, and document clustering analysis.

### 2.1. Software Selection and Parameter Definition

Chaomei Chen developed CiteSpace, which uses Java for data visualization analysis [32]. It generates co-citation network maps by processing and analyzing large-scale bibliometric data. Researchers have used CiteSpace to review literature in different academic fields, including areas related to aging. For example, Liao Jianfeng et al. [36] analyzed the literature on elderly smart homes using CiteSpace, revealing research hotspots and future directions. In addition, Chen Zhe et al. [34] collected literature on elderly care and used CiteSpace to explore knowledge gaps and future trends. Combining bibliometrics and CiteSpace offers researchers a convenient and efficient tool to precisely identify research hotspots and future development directions on specific topics [37].

VOSViewer is a robust network analysis and visualization tool developed by the Centre for Science and Technology Studies at Leiden University in the Netherlands. It is designed for handling large datasets and can create and analyze complex network maps, especially in bibliometrics and knowledge mapping [33]. Moreover, VOSViewer is frequently used in fields related to aging. For instance Yenişehir Semiha et al. [38] used VOSViewer to construct a knowledge map exploring in-depth the application and development of artificial intelligence in fall prevention among older adults. Additionally, Hong Yi-Kyung et al. [39] performed a co-occurrence visualization analysis of keywords and authors using VOSViewer to elucidate global research trends in smart homes for older adults. In summary, combining VOSViewer with CiteSpace can provide a comprehensive tool for analyzing bibliographic data [34].

This paper uses CiteSpace software (version 6.3.R3) and VOSViewer software (version 1.6.20) to comprehensively analyze the existing research on the informatization of community elderly care. Furthermore, the data is analyzed and visualized via bibliometric analysis using Microsoft Excel 2019, Charticulator, and Scimago Graphica. Charticulator is an open-source tool for custom chart design [40], whereas Scimago Graphica is a free, user-friendly software for quickly generating basic charts [41].

Furthermore, “nodes” and “links” are two fundamental elements when constructing knowledge maps. The size of a node typically represents its influence or intensity, while the thickness of a link indicates the closeness of the relationship between nodes. This article also introduces the parameter “k” to highlight the main content of the spectrum. Only when the node value of a country, institution, or author exceeds “k” will the node be displayed on the map. This approach aims to more effectively identify and illustrate the critical nodes and relationships within research collaboration networks.

### 2.2. Data Collection and Processing

The Web of Science Core Collection (WoSCC) is a comprehensive academic literature database that provides extensive and high-quality document data for scientific research [42]. Researchers use WoSCC as a reference data source for bibliometric analysis [43]. Therefore, this study collected data through WoSCC and used CiteSpace and VOSViewer to process the data for further bibliometric analysis.

Furthermore, this study conducted a search using WoSCC, with the specific search strategy shown in Table 1. The search included the Science Citation Index Expanded (SCI-EXPANDED), Social Sciences Citation Index (SSCI), and Arts & Humanities Citation Index (A&HCI). The time frame was restricted from 2004 to 2024, yielding 1023 articles. Then, only English papers were selected, excluding 129 documents of other types. After manual screening, 433 documents with unrelated topics were excluded. For instance, documents lacking seniors, as well as those lacking content on community services and those missing information technology application content. Ultimately, a total of 461 documents were included in the analysis.

### 2.3. Descriptive Statistical Analysis

#### 2.3.1. Quantitative Analysis

**Figure 2:**
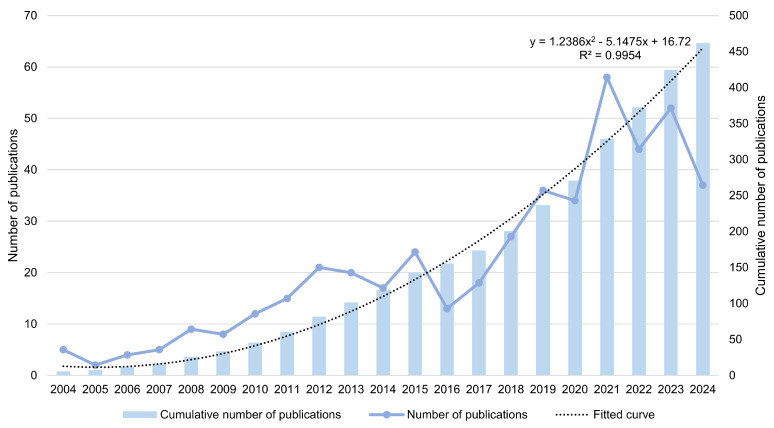
Distribution of publication years of articles related to research from 2004 to 2024.

This study examines the publication years of 461 articles about the informatization of community elderly care services, as illustrated in Figure 2. From 2004 to 2010, the number of publications grew slowly but steadily. During this period, with the gradual development of computer and medical instrument technologies [44], the conceptualization and practice of empowering community services for the elderly with information began to take off [45]. From 2010 to 2021, the number of publications increased significantly. During this stage, the issue of population aging became gradually more severe. Considering China as an example, the elderly dependency ratio rose from 12.7% in 2012 to 20.8% in 2021, an increase of 8.1%, which drove the rapid development of elderly care services [36]. The trend in publications continued to grow until 2024. The primary reason for this phenomenon is the rapid development of IT, which has facilitated the creation of innovative solutions for elderly care [46,47]. Secondly, the global demographic shift towards an aging population has increased the demand for service models that improve the quality of life for older people [48,49]. Furthermore, many countries have started emphasizing community elderly care services, promoting research in this domain [50].

#### 2.3.2. Category Analysis

**Figure 3:**
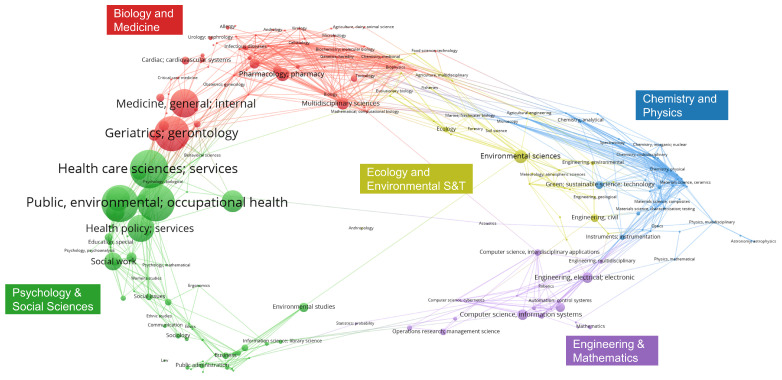
Visual analysis of research categories on information technology-enabled elderly care community services.

Category analysis elucidates the spectrum of disciplines encompassed within a particular knowledge domain. Through the analysis of 461 pieces of literature, we derived Figure 3. This study found that environmental science and health sciences are the primary disciplines in this field, holding a significant position. The most cited article in environmental science is Liu Sunwei et al.’s “Two-Step Floating Catchment Area Model-Based Evaluation of Community Care Facilities’ Spatial Accessibility in Xi’an, China” [51]. In health sciences, health care sciences services is the most studied topic, comprising 86 related articles, the most prominent of which is by Namkee G Choi and DiNitto Diana M, titled “Internet Use Among Older Adults: Association With Health Needs, Psychological Capital, and Social Capital” [52]. Furthermore, engineering and electrical and electronic engineering are important research disciplines, indicating that research in this field primarily focuses on environmental health, healthcare services, and interdisciplinary studies in engineering and electronics. Moreover, computer science, information systems, artificial intelligence, and social sciences have also contributed to research in this field, collectively driving the development of this domain.

#### 2.3.3. Journal Analysis

The journal analysis systematically evaluates metrics such as publications, citation rates, impact factors, and research fields to elucidate the academic impact, research quality, and publication trends of journals. Table 2 depicts that the United Kingdom’s BMJ Open has the highest number of publications, with 24 articles. This is followed by the United Kingdom’s BMC Health Services Research and BMC Geometry, with 19 and 17 articles, respectively, indicating that researchers in the field favor these journals and have made significant contributions to the area. Of the top 10 journals by publication volume, 5 are based in the UK and 3 in the USA, indicating that these countries are highly active in research on elder care community services and are leaders globally in publication volume in this domain.

## 3. Cooperation Analysis

Cooperation analysis can identify the most active countries, institutions, and authors in a specific research field and their collaboration patterns [53]. The co-country analysis in this section identifies the major contributing countries to applying information empowerment in elderly community services from a macro perspective. Examining collaborative institutions provides a meso perspective on prominent research organizations. Furthermore, collaboration analysis at the micro level identifies the key researchers in this domain.

### 3.1. Country Cooperation

**Figure 4:**
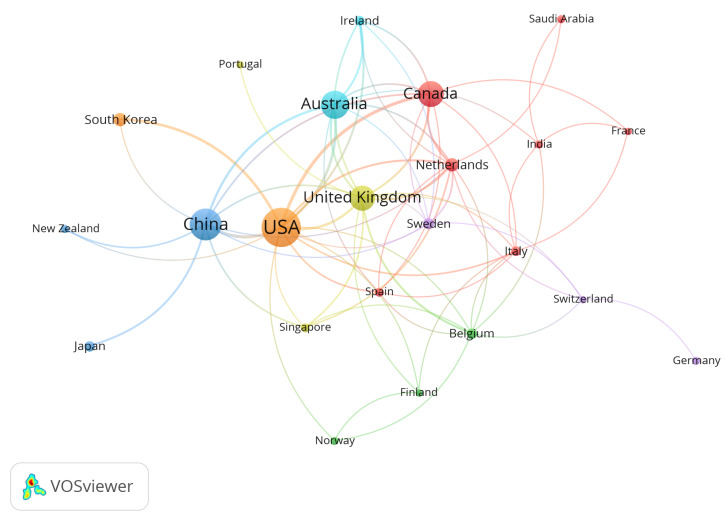
National cooperation network diagram.

The analysis of international collaborations revealed the distribution patterns of publications among different countries, offering a quantitative framework for understanding global academic exchanges [53]. Figure 4 uses VOSViewer to analyze international collaborations, showcasing 23 countries with fewer than five publications. Close collaborations among the countries are evident, showing a team-based cooperation scenario. Figure 4 illustrates that the total collaborations of the USA with other countries significantly surpass those among other countries. Notably, frequent collaborations between the USA and China, the USA and Canada, and the USA and South Korea indicate that the USA is an important hub in this field and has made significant contributions.

A total of 49 countries have contributed to this field globally. This study selected the top ten countries by publication volume and created Table 3. The USA has the highest number of publications, with 129 papers, while China and Australia rank second and third with 86 and 68 papers, respectively. A potential reason for the high publication volume in these countries is the policy support and funding from government institutions such as the US Department of Veterans Affairs [4,54], Chinese government departments [55,56], and Australian community-age care organizations [57,58]. Through data analysis, this study found that the average citation frequency per article is highest in the Netherlands, followed by the United Kingdom, the USA, and Belgium. Moreover, the study collected the H-index data of the top ten countries. The results indicated that the USA possesses the highest H-index, signifying its predominant academic influence in this domain. The research status of these countries deserves attention from researchers.

### 3.2. Institution Cooperation

**Figure 5:**
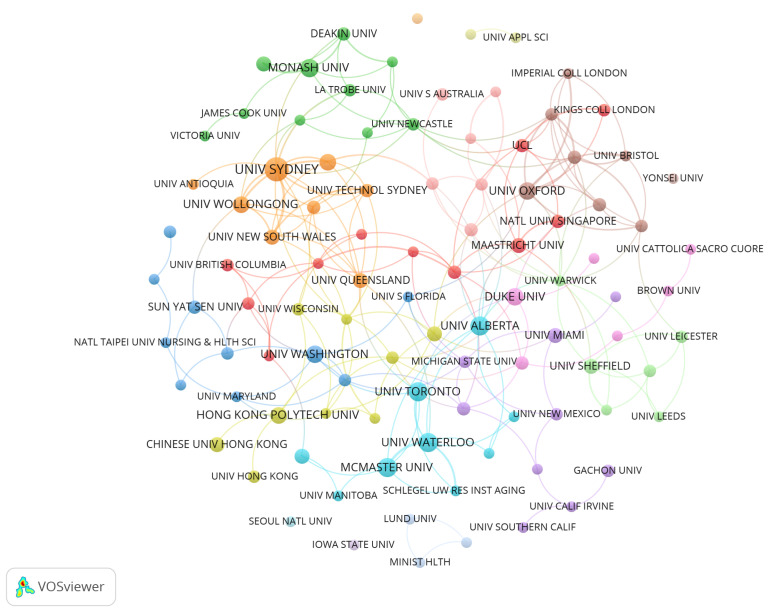
Institutional cooperation network diagram.

The analysis of institutional collaboration seeks to comprehensively understand collaboration mechanisms, evaluate collaboration potential, enhance resource allocation, attain mutual benefits, and foster the collective advancement of all parties involved [53]. This study uses VOSViewer to analyze institutional data (see Figure 5). Figure 5 shows 100 institutions with at least three publications each. Most of these institutions have close collaborations, which benefits development in this domain. Moreover, regional institutional collaboration clusters such as Hong Kong Polytechnic University, Chinese University of Hong Kong, and University of Hong Kong appear in multiple regions, facilitating knowledge and resource sharing within the region. Furthermore, the cooperating institutions are involved in various disciplines such as medicine [59], health sciences [60], and IT [24], reflecting that information empowerment of elderly care community services is a multidisciplinary research domain.

There are 1002 institutions dedicated to this field. Table 4 lists the top ten institutions by the number of publications. The University of Sydney has the most publications, totaling 16. Its primary research direction is the informatization of elderly health services [5,61]. Following this are the University of California System, University of London, US Department of Veterans Affairs, and Veterans Health Administration VHA, each with 13 publications. The institution ranked second primarily for research on elderly healthcare [62,63]. The third-ranked institution focuses on the development of health technology for older people [64,65]. The fourth and fifth institutions investigate elderly home care [4,66] and digital elderly care [67,68]. Furthermore, the University of London has the highest H-index and average citation frequency per article, indicating its leading role in research within this field. The institution’s most influential article is “Why is it difficult to implement e-health initiatives? A qualitative study” [64], which confirms the ongoing research significance of implementers’ understanding of barriers and facilitators to successful implementation, emphasizing the critical role of normalization process theory in elucidating implementation changes and informing future implementation strategies.

### 3.3. Author Cooperation

**Figure 6:**
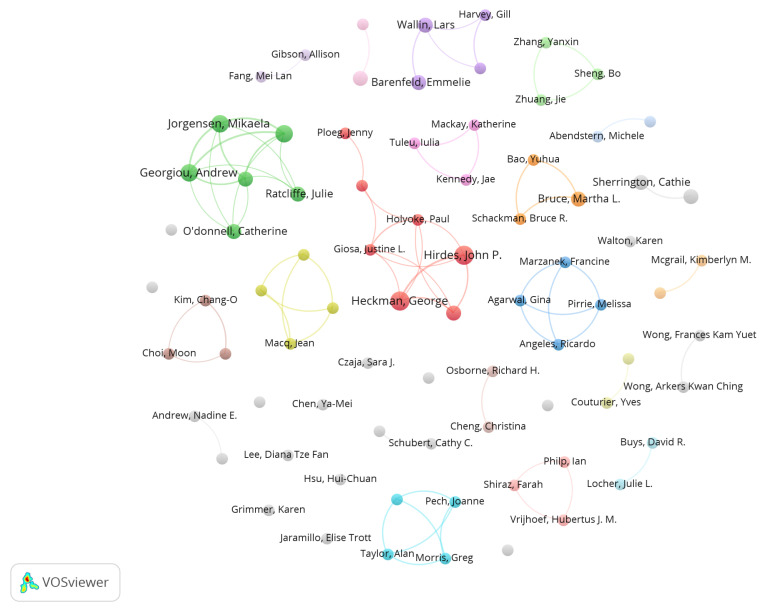
Author cooperation network diagram.

The analysis of author collaboration seeks to elucidate the structure of academic networks, collaboration patterns, and knowledge flow by studying the cooperative relationships between authors [53]. The VOSViewer tool was utilized to analyze 79 authors, each with at least two publications, resulting in the author collaboration network map shown in Figure 6. The research indicated that authors like John P. Hirdes and George Heckman possess more publications in this domain. Some authors formed specific research groups, such as a team centered around Hirdes John P. and Heckman George, primarily focusing on community healthcare for older adults [69,70]. Moreover, the team centered around Andrew Georgiou and Mikaela Jorgensen focuses on community care for older persons [71,72]. Overall, the author’s collaboration network map has low connectivity density, with limited cooperation between research groups and some independent authors. This dispersed collaboration model is not conducive to the development of the field.

Table 5 presents statistics based on research conducted by 2599 authors in this domain, enumerating the top 10 authors by publication volume. Among them, John P. Hirdes and George Heckman possess the highest number of publications, with five articles each. Their most cited joint article is “Caregiver Status Affects Medication Adherence among Older Home Care Clients with Heart Failure” [73], which primarily examines the influence of caregiver status on medication adherence among elderly heart failure patients receiving home care. This study is crucial for community elderly care services in improving medication management and optimizing caregiver assistance. Other authors with a relatively high number of publications include Andrew Georgiou, Mikaela Jorgensen, and Johanna Westbrook, each having published four articles. Andrew Georgiou and Mikaela Jorgensen concentrated their research on community care for older people [71,72]. Conversely, Johanna Westbrook focused on the informatization evaluation of community services for older people [74,75]. The author with the highest average citations per article is Catherine O’Donnell, whose most cited article is “Why is it difficult to implement e-health initiatives? A qualitative study” [64]. Catherine O’Donnell’s primary research area is e-health [64,72,76], which corresponds with multiple trends, such as digital transformation and data democratization [77,78], artificial intelligence and machine learning [77,79], digital health technology [80], and human-centered design [81]. These trends suggest that e-health is advancing towards a more interconnected, personalized, and secure future, deserving the attention of researchers.

## 4. Keyword Analysis

Keyword analysis reveals the co-occurrence and emergence relationships among vocabulary, helping researchers understand the research hotspots and thematic structure changes in a disciplinary field [82]. This section analyzes the co-occurrence frequency and emergence of keywords in the literature using statistical data and graphical methods, thereby uncovering research trends and the knowledge structure in this field.

### 4.1. Keyword Co-Occurrence

**Figure 7:**
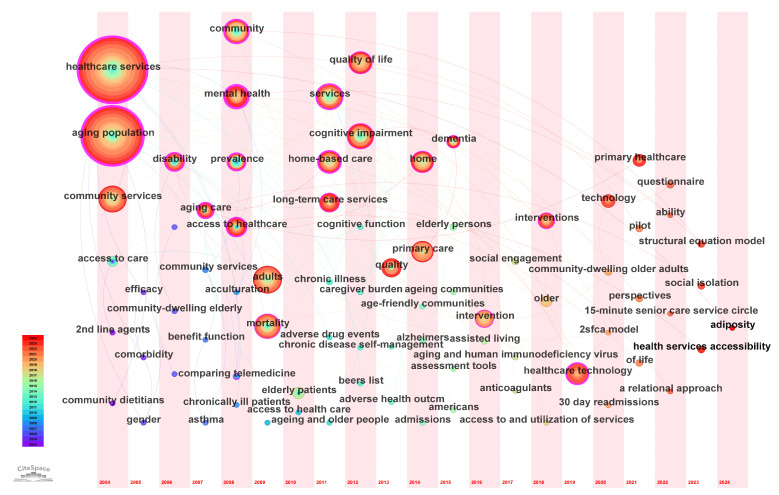
Keyword time zone graph.

Keyword co-occurrence analysis is primarily used to identify and analyze keywords present in academic literature, elucidating the connections between research topics and hotspots [83]. Based on the g-index, this study set a scaling factor (k = 15) to extract keywords from the 461 included documents, yielding 272. Keywords were then merged and deleted accordingly. CiteSpace uses the g-index as a selection criterion for selecting nodes, adjusting the scaling factor (k) to determine inclusion or exclusion. After processing, we identified 81 keywords and illustrated the final results in Figure 7.

Figure 7 illustrates that some nodes are surrounded by a purple band, signifying that these nodes possess a betweenness centrality greater than 0.1 and play a crucial bridging role in the network. The keywords “Healthcare services” and “Aging population” have the highest betweenness centrality. “Healthcare services” denote the organized medical treatment, preventive care, and health education provided to individuals and communities by healthcare professionals and auxiliary professionals [84,85]. “Aging population” denotes the increasing proportion of elderly individuals in a population structure and the significant impacts of this trend on the socio-economic environment, health systems, labor markets, family structure, and intergenerational relationships [86,87,88]. “Community” refers to a group of individuals or collectives within a specified area who possess shared characteristics, interests, or objectives, encompassing social morphology, organizational patterns, and modes of interpersonal interaction while addressing members’ daily needs and interests through public resources [89,90]. “Services” denote activities and processes organized to meet customer requirements, primarily referring to elderly care-related services and processes [91,92].

Figure 7 illustrates the evolution of research hotspots in this domain. “Healthcare services”, “Aging population”, and “Community services” have consistently been the primary topics discussed in this domain. Other themes revolve around these three central topics, necessitating continuous focus from researchers on these subjects. Important keywords derived from these three main topics encompass “Structural equation model”, “Social isolation”, and “Health services accessibility”, which constitute the core contents of current research. The “structural equation model (SEM)” is a multivariate statistical analysis technique used to represent causal relationships between variables, indicating the strength of these relationships through path coefficients. It addresses measurement errors in observed variables, facilitating a more precise measurement of theoretical constructs of interest [93]. “Social isolation” refers to the lack of social contact, encompassing both an objective lack of social activities and networks as well as a subjective experience of loneliness and disconnection from society [94]. “Health services accessibility” refers to the ability of individuals or groups to acquire essential medical services promptly when required. This concept encompasses multiple dimensions, such as availability, reachability, affordability, acceptability, and appropriateness of services, which influence health outcomes and social equity [95].

### 4.2. Keyword Emergence

**Figure 8:**
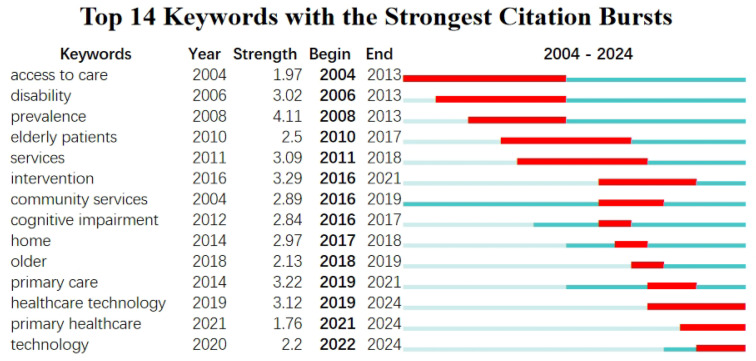
Keyword emergence analysis.

Keyword burst analysis is a bibliometric method to identify and monitor fluctuations in research focal points and trends within a particular domain [23]. Figure 8 depicts the citation burst strength of 14 keywords in the academic literature from 2004 to 2024. The red lines in the figure indicate the citation burst period of keywords, during which the citation frequency for these keywords significantly increases, suggesting that the topic has garnered considerable attention in the academic community. The dark blue line indicates the duration from the inception to the conclusion of the keyword, signifying the commencement and termination of the citation burst period. The distribution of the red and blue lines allows for segmenting keyword bursts into three distinct stages.

The first phase, spanning from 2004 to 2011, marked the initial exploratory stage of IT-enabled community services for older persons. Despite the relatively few documents and studies during this period, the potential of IT in addressing the challenges of population aging was evident. The research hotspots, such as “access to care”, “disability”, “prevalence”, “elderly patients”, and “services”, laid the foundation for future advancements in the field.

Among them, the first three keywords indicate that during this period, research on the accessibility of healthcare for older persons, the rights of elderly disabled persons, and disease prevalence has received significant attention [9,18,96]. In particular, the high intensity and duration of the keyword “prevalence” demonstrate the active nature of elderly epidemiology research during this time [96]. Research on “access to care” focuses on the accessibility of healthcare services, especially in resource-limited settings [18]. “Disability” mainly concentrates on the social participation of elderly disabled persons, constructing barrier-free environments, and protecting their rights [9]. Additionally, “elderly patients” and “services” reflect the emphasis on health issues of older adults, service science, and service quality improvement. Research on “elderly patients” primarily involves the health needs, chronic disease management, and elderly care services of senior patients [6]. “Services” pertains to service quality improvement, service innovation, and the application of service science in various fields [91,92].

The second phase, spanning from 2012 to 2018, denotes a significant enhancement in community services for older people facilitated by IT. With technological advancements and policy support, smart community elder care services started receiving widespread attention from society and academia, significantly increasing research publications. Moreover, smart elderly care emerged, and elder care service models evolved towards intelligence, technology, and humanization. The development of elder care information systems and smart nursing home facilities has become increasingly significant. During this phase, research hotspots shifted to “intervention”, “cognitive impairment”, “home”, “community services”, and “older”.

The term “intervention” indicates the enhancement of research in comprehensive elderly and digital health interventions, including comprehensive elderly intervention measures, digital health interventions, and preventive strategies [97]. “Cognitive impairment” primarily focuses on diagnosing, treating, and managing cognitive disorders in older adults, reflecting increased academic attention to cognitive disorders and neurodegenerative diseases [98]. “Home” primarily examines the development of home healthcare, telemedicine, and home care services, highlighting the rise of home-based elder care services during this phase [99]. Furthermore, “community services” garnered attention during this period and continued until 2019, underscoring their significance in enhancing social welfare and health standards [100]. Zhou Junshan et al. [101] emphasized the crucial role of community services in providing necessary support and care for older people. Moreover, “older” signifies the increasing importance of research about geriatric health, eldercare services, and geriatric medicine [102].

The third stage spans from 2019 to 2024, during which IT has empowered the elderly care community service sector to embark on a new era of rapid development. At this stage, the relevant fields have developed rapidly, with the continuous emergence of technological and business model innovations, resulting in products and services that exhibit diversification and personalization [23]. Many countries worldwide have increased their support and investment in the smart elderly care sector, fostering innovation and development of the smart senior care industry [103,104]. At this stage, “healthcare technology”, “primary healthcare”, “primary care”, and “technology” have emerged as research hotspots.

Research on “primary care” predominantly examines the quality, accessibility, and efficiency of primary healthcare services, indicating that primary care constitutes the foundation of the eldercare health system and is crucial for improving overall healthcare outcomes [84]. Studies on “healthcare technology” and “technology” indicate that the application of eldercare medical technology and other innovations in the medical field is rapidly growing. “Healthcare technology” refers to the latest advancements in medical technology, encompassing telemedicine, electronic health records, and medical devices [105]. For example, Papa Armando et al. [106] explored the application of intelligent medical devices in health monitoring, emphasizing their role in improving health management and reducing medical costs via remote monitoring. Conversely, “technology”, refers to the utilization of technological advancements in various sectors of healthcare and eldercare, encompassing artificial intelligence, big data, and the Internet of Things [84]. Furthermore, research on “primary healthcare” has shown a surge in significance from 2021 to 2024, reflecting the growing importance of primary healthcare systems in global health policy [107].

During the development process of IT empowering elderly care community services, the changes in keywords reflect the research focus and developmental trends at each stage. During the exploratory stage from 2004 to 2011, research hotspots were concentrated on “access to care”, “disability”, “prevalence”, “elderly patients”, and “services”, establishing the foundation for subsequent development. During the continuous expansion from 2012 to 2018, the focus shifted to “intervention”, “cognitive impairment”, “home”, “community services”, and “older”, indicating a transition in elderly care service models towards intelligence, technology, and humanization. During the rapid development era from 2019 to 2024, research hotspots further focused on “healthcare technology”, “primary healthcare”, “primary care”, and “technology”, while community elderly care services evolved towards diversification and personalization. The changes across the three stages illustrate the diversity and complexity of research in information-enabled elderly care community services while indicating that the smart elderly care service model is continuously innovated and refined in response to evolving elderly care challenges and technological progress.

## 5. Co-Citation Analysis

Co-citation analysis is a method for assessing the impact of the academic literature. It identifies the research hotspots by counting the frequency of citations for journals, authors, and documents [108]. Among them, document clustering analysis can be used to identify the similarities and differences in research topics. This section uses citation analysis to identify significant research in IT-enabled community services for older people, offering references for academic evaluation and research direction.

### 5.1. Journal Citation

**Figure 9:**
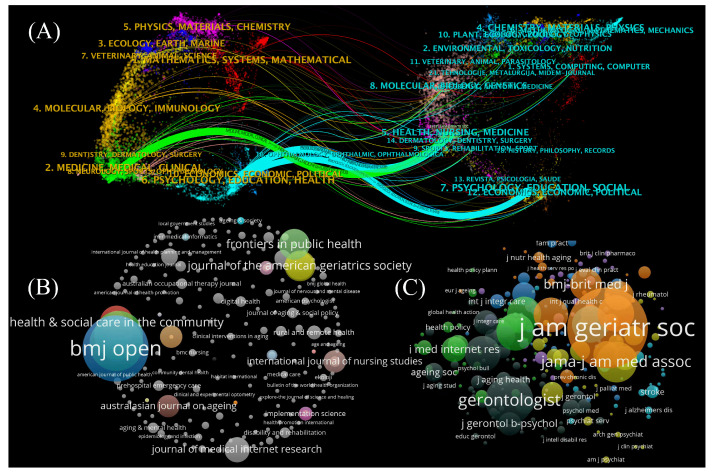
(**A**) Double graph overlay. (**B**) Citing journals. (**C**) Cited journals.

Significant journals are essential in this domain. The nodes in Figure 9 represent journals with a minimum of 5 published articles (18 in total). Figure 9 describes the dual-map overlay between citing and cited journals of 461 related articles. It illustrates the citation relationships within the research domain, cross-integration, and mutual influence among different disciplines.

Figure 9A illustrates that journals in clinical medicine, psychology, education, and health sciences primarily cite research articles published in journals with focuses such as healthcare and medicine. The situation indicates that in IT empowering community services for older people, the research focus of these fields is highly relevant and complementary, which is characterized by frequent academic exchanges and collaborations. Moreover, research articles published in psychology, education, and sociology journals are often cited by journals in psychology, education, and health sciences, confirming the close connection and mutual penetration between these disciplines. Figure 9B,C provide detailed information on citing and cited journals, along with overlay maps illustrating these connections.

Figure 9B depicts that journals such as *Health and Social Care in the Community*, *BMJ Open*, *Frontiers in Public Health*, and *Journal of the American Geriatrics Society* (*J AM GERIATR SOC*) often cite articles in this field, suggesting that articles on IT empowering community services for older people receive widespread attention in these journals. Figure 9C shows that articles in this domain often cite articles in journals such as the *J AM GERIATR SOC*, *JAMA-Journal of the American Medical Association* (*JAMA-J AM MED ASSOC*), and *The Gerontologist*. Figure 9B,C suggest that these journals facilitate a deeper understanding of the disciplinary foundations and theoretical sources of articles in this field.

Table 6 indicates that the *J AM GERIATR SOC* from the USA is cited 417 times, securing the first position. This journal focuses on research in geriatrics [66], offering a robust theoretical foundation for enhancing community services for older people via IT. *The Gerontologist* and *JAMA-J AM MED ASSOC*, both from the USA, followed with citation frequencies of 231 and 193, respectively. *The Gerontologist* primarily focuses on the biological changes in older people [109] and involves research on social, psychological, and economic factors in the aging process [60,110]. Conversely, *JAMA-J AM MED ASSOC* focuses on comprehensive medicine, with its multidisciplinary medical research significantly contributing to developing health management strategies for older people [24,111].

### 5.2. Author Citation

**Figure 10:**
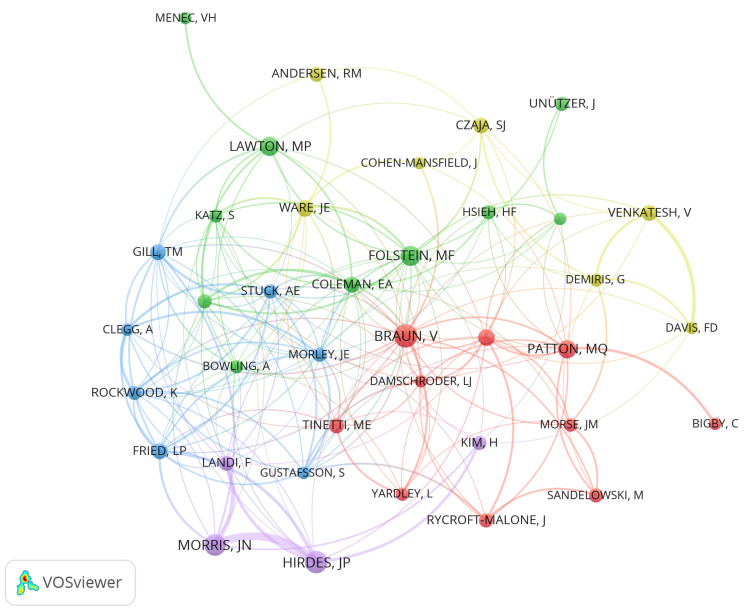
Author citations.

Author citation analysis elucidates the relationships and impact of various authors in academic research, facilitating the identification of key scholars within a discipline, prospective collaboration avenues, and trends in interdisciplinary research [108]. This paper uses the VOSViewer tool to construct a co-citation network of authors, with Figure 10 displaying authors cited at least 10 times (a total of 38). Virginia Braun’s node is the most prominent, possessing the highest citation frequency. John P. Hirdes and John N. Morris are ranked second and third, respectively, and are members of the same research team, as evidenced by the thicker line indicating their strong collaborative relationship. Furthermore, Marshal F. Folstein and M. Powell Lawton are part of the same research group, albeit their association is comparatively weak. These findings facilitate the identification of key scholars, potential collaboration opportunities, and trends of interdisciplinarity within the research domain.

Table 7 illustrates that the most frequently cited author is Virginia Braun, who focuses on the analysis of psychological themes such as “epistemology”, “qualitative psychology”, and “thematic analysis” [112]. The most cited article by Virginia Braun is “Using Thematic Analysis in Psychology”, a seminal guide on thematic analysis in psychology [112]. John P. Hirdes, the most prolific author in this field, has a highly cited article, “Development of a minimum data set-based depression rating scale for use in nursing homes”,which is a significant source for mental health services for older people [113]. John N. Morris focused on research on “chronic cognitive impairment”, “continuum of care”, and “scale development”, and has authored the highly cited article “MDS Cognitive Performance Scale©” [69,114]. Furthermore, Marshal F. Folstein primarily investigated subjects related to mental state assessment, such as the “Cognitive State Grading Method”, “Clinical Assessment Tool”, and “Neuropsychological Evaluation Technique” [115]; his most highly cited article is “Mini-mental State: A Practical Method for Grading the Cognitive State of Patients for the Clinician” [115]. M. Powell Lawton performed research on “older adults” and “instrumental activities of daily living” [116] and his most frequently cited article is “Assessment of Older People: Self-Maintaining and Instrumental Activities of Daily Living” [116].

### 5.3. Document Citation

**Figure 11:**
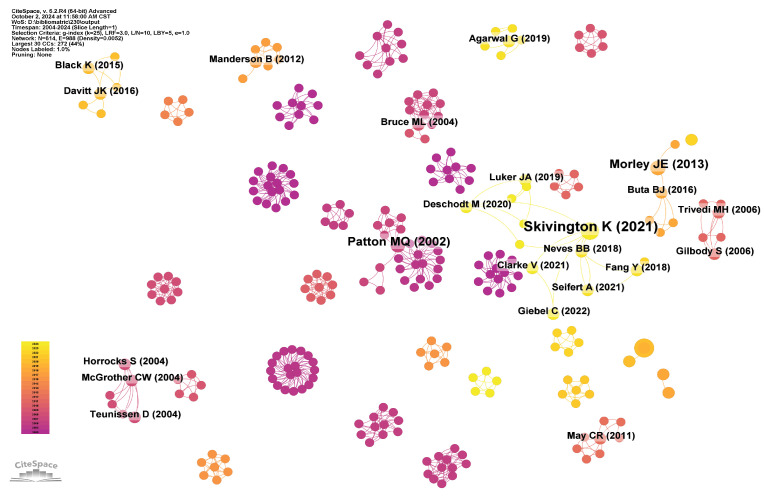
Documents cited together.

Document citation analysis indicates the academic influence of studies, research quality, and recognition within the academic community and is a significant metric for assessing scholars’ academic contributions [117]. This study uses citations within one year as a time unit, specifically on article published from 2004 to 2024. The citation reference network comprises 1427 nodes and 6492 links (Figure 11). The analysis of Figure 11 and Table 8 indicates that the article with the highest co-citation frequency is “A New Framework for Developing and Evaluating Complex Interventions: update of Medical Research Council Guidance”, published in the *BMJ-British Medical Journal*, authored by Kathryn Skivington [118]. This article revises the guidelines of the British Medical Research Council for the development and assessment of complex interventions and introduces a novel framework. The new framework facilitates the systematic development and evaluation of complex interventions for older people, enhancing the effectiveness and quality of their services.

The second-ranked study is “Global Estimates of the Need for Rehabilitation based on the Global Burden of Disease Study 2019: A Systematic Analysis for the Global Burden of Disease Study 2019”, published in *The Lancet* [119]. This study assesses the global need for rehabilitation. It suggests that service providers should focus on the development of the informatization of rehabilitation services to meet the growing rehabilitation needs of older people.

Additionally, the study “Frailty Consensus: A Call to Action” by John E. Morley, published in *The Journal of the American Medical Directors Association*, is ranked third [120]. This study primarily explores the concept and definition of frailty and its relationship with aging and chronic diseases. It emphasizes the importance of frailty as an emerging research and clinical paradigm in geriatric medicine. It points out the necessity of using informationization methods to monitor, assess, and intervene in frailty and slow the progression of frailty in older adults.

### 5.4. Cluster Analysis

**Figure 12:**
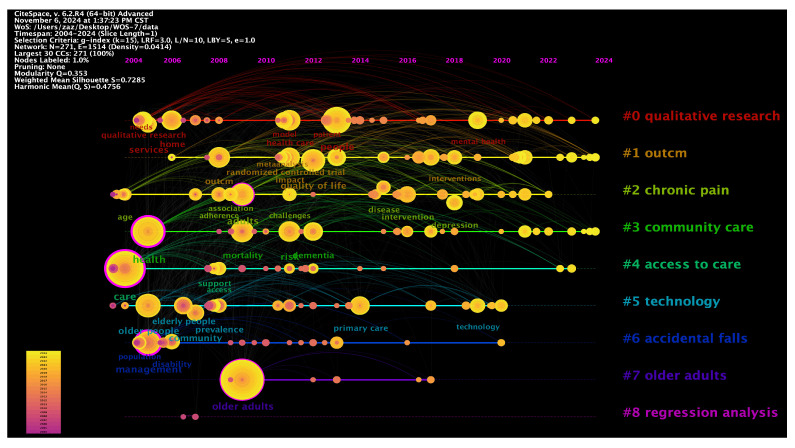
Document clustering.

Cluster analysis can quickly identify research trends within a field, reveal knowledge structures, and facilitate academic exchange [117]. Figure 12 depicts the cluster analysis results of 461 documents, from which 271 keywords were extracted. This study uses two years as a segment, merging similar clusters from different periods and counting their citations to ultimately identify nine main clusters. Among them, Cluster 0 and Cluster 8 pertain to research methods within other clusters, Cluster 1 relates to result evaluation methods within other clusters, whereas Clusters 2 and 6 involve specific issues concerning older adults. Therefore, this study excludes these five clusters and provides a detailed discussion of the remaining four clusters.

Among these clusters, the community care cluster has the highest number of citations, indicating it is a popular topic in this field. Since its inception in 2004, it has gradually become a key component of elderly community services. The increasing global aging population and the critical shortage of caregivers have led to increased scrutiny of access to care by researchers. In recent years, ongoing research in this domain has revealed that technology clusters for elderly services are consistently improving. The following is a specific discussion of each cluster.

(1) Community care
  #3 Community care is an essential domain that entails improving the quality of life for older people through diverse technological methods, allowing them to enjoy their later years within a familiar community setting. Gustafson and colleagues investigated the influence of information and communication technology (ICT) on the quality of life of older people, examining the potential application of ICT in community care via a randomized controlled trial [5]. Furthermore, Taylor and colleagues concentrated on home telemedicine video conferencing, analyzing the perceptions and efficacy of this technology in delivering community care services [7]. Northwood and colleagues used a group concept mapping methodology to investigate the application of electronic health tools (interRAI Check-Up Self Report) in facilitating the integrated health and social care of older people and their caregivers within the community [84]. This tool can integrate health information, enhance care efficiency, and, via multidisciplinary team collaboration, deliver more comprehensive and compassionate care services for older adults. Rahmawati Riana et al. demonstrated the function of community health workers in managing hypertension in older people [128], emphasizing the significance of community care in managing chronic diseases.
  While IT has helped communities address various elderly care issues, numerous challenges in caring for older adults persist due to social factors. For instance, considerable challenges arise in domains such as the prevalence and acceptance of technology [10], cross-cultural differences, and service compatibility [129]. To effectively address these challenges, it is essential to enhance the user experience design of IT by thoroughly considering the cognitive capacities, cultural contexts, and usage patterns of older individuals, thereby improving their satisfaction and the efficacy of elderly care services.
(2) Access to care
  Improving the accessibility of medical and nursing services for older people (#4 Access to care) is a core issue. Numerous studies have explored this topic from different perspectives, and some of these studies have integrated IT to provide more convenient and efficient health services for older people. Soran and others indicated that computerized telephone monitoring systems are superior to conventional care in reducing healthcare costs for elderly patients with heart failure [6]. Moreover, Chiu Kuang and colleagues in Taiwan further confirmed the feasibility of remote monitoring of the health status of older people at home [8]. These studies enhance the ability of older people to access medical services, reduce healthcare costs, and improve the efficiency of medical resource utilization.
  Despite the increasing number of studies on the accessibility of medical and nursing services, there are still many areas that need improvement. For instance, Gao Li and others have explored the inadequacies in the integration of health resources for the elderly [130]. Meanwhile, Hogeveen Sophie and colleagues investigated the challenges faced by the elderly in accessing healthcare services [131]. These studies indicate that the elderly still face a series of issues regarding the accessibility of healthcare services, including insufficient service integration and coordination, low acceptance of technology, a significant digital divide, uneven resource distribution, and variations in service quality. To effectively address these challenges, it is urgently necessary for the government, society, and service providers to strengthen cooperation and promote the application of information technology in elderly community services, thereby facilitating the continuous development and improvement of services.
(3) Technology
  The application of technology is a key driver for upgrading and optimizing services. Among the various technologies, telemedicine, intelligent assistance systems, and digital tools are particularly significant. These technologies provide more comprehensive and personalized services for older adults through various methods. Chiu Kuang et al. and Taylor et al. demonstrated that remote monitoring technology can effectively monitor and care for the health status of older people [7,130]. Both studies demonstrated that this technology enhances the efficiency of medical services and significantly improves the quality of life for older individuals. Furthermore, the study by Fan et al. shows that compared to traditional in-person medical consultations, health chatbots improve the efficiency of self-diagnosis among older persons by 40% [132]. This research provides empirical evidence for the clinical implementation of health chatbots. Regarding intelligent assistance systems, Gustafson et al. demonstrated the effectiveness and comprehensiveness of information and communication technology (ICT) in improving the quality of life for older people [5]. Moreover, Northwood et al. used electronic health tools to integrate health and social care information, enhancing collaboration among interdisciplinary teams to deliver more comprehensive and compassionate care for older people within the community [84]. In the field of artificial intelligence applications, a study by Ghosh et al. proposed the “FEEL” framework, which achieves federated learning through edge-IoMT devices, reducing the risk of data breaches by 89%. This framework provides a secure foundation for multi-institutional collaborative community health networks [133]. Moreover, Lima et al. provided personalized companionship through multimodal emotion recognition, significantly alleviating anxiety in dementia patients [134]. Furthermore, Wilmink et al. found that an AI-driven health platform, combined with wearable devices, can reduce the risk of falls by 32%, providing key clinical evidence for technology-based preventive interventions [135]. These digital tools improve care efficiency and ensure that empathy in care is preserved by standardizing assessment technologies during their implementation.
  Despite the significant potential these technologies show in eldercare community services, many challenges need to be addressed. Wong et al. and Fan et al. indicated that the high complexity of system development results in a lack of user-friendliness and interface accessibility, leading to limited acceptance among older people [132,136]. Moreover, Vergouw et al. noted that older individuals often encounter difficulties when using applications and lack access to professional guidance [137]. Additionally, the research by Wilmink et al. also indicates that issues with the comfort of wearable devices can lead users to discontinue their use [135]. These studies illustrate that future technology needs to balance precise monitoring and user experience, continuously enhancing the informational empowerment and service quality of eldercare community services through technological innovation and service optimization.
(4) Older adults
  #7 Research concerning older adults has attracted significant attention. These studies aim to improve the quality of life for older people, augment their autonomy, and enhance their social interaction abilities. Wong and colleagues examined the technological acceptance of the Intelligent Comprehensive Interactive Care (ICIC) system among older people [136]. The research indicated that older people strongly accepted the system, suggesting its potential utility in community elder care services. Furthermore, Gell Nancy M. and associates examined technology usage trends among older people with and without disabilities [109]. This research offers significant references for developing technological products and services tailored to the varied needs of older people. Kim and colleagues developed a healthcare service system tailored for older adults [138]. Their research demonstrated that the system markedly enhanced the accessibility and quality of healthcare services for older people. Furthermore, Askari Marjan and colleagues concentrated on the usage intentions of older Dutch people concerning medical applications [139]. This research provides significant insights for developing medical applications tailored to the requirements of older people in the Netherlands.
  Although there have been some advancements in IT to address the challenges older people face in recent years, several issues remain insufficiently addressed. Jamerson et al. noted that older adults encounter various challenges in medication management attributable to cognitive decline [140]. Furthermore, Kim and associates indicated that older people face challenges adapting to and embracing new technological paradigms [138]. Furthermore, the integration of information technology with elder care services requires careful consideration of its profound ethical and social implications. Firstly, the collection of sensitive health data poses risks to privacy and data security [141,142]. Secondly, the digital divide caused by disparities in digital literacy and economic resources may exacerbate inequality and social exclusion among the elderly population [143,144]. Excessive reliance on technology may also weaken the necessary interpersonal interactions within care services [145]. In addition, ensuring the autonomy and informed consent of older adults when their data is used presents a significant challenge, particularly for those with cognitive impairments [146,147]. Therefore, in parallel with technological innovation, establishing a robust ethical framework, adopting inclusive design principles, and engaging in ongoing social dialogue is crucial for the equitable and responsible development of information technology in elder care services.

## 6. Future Research Directions

**Figure 13:**
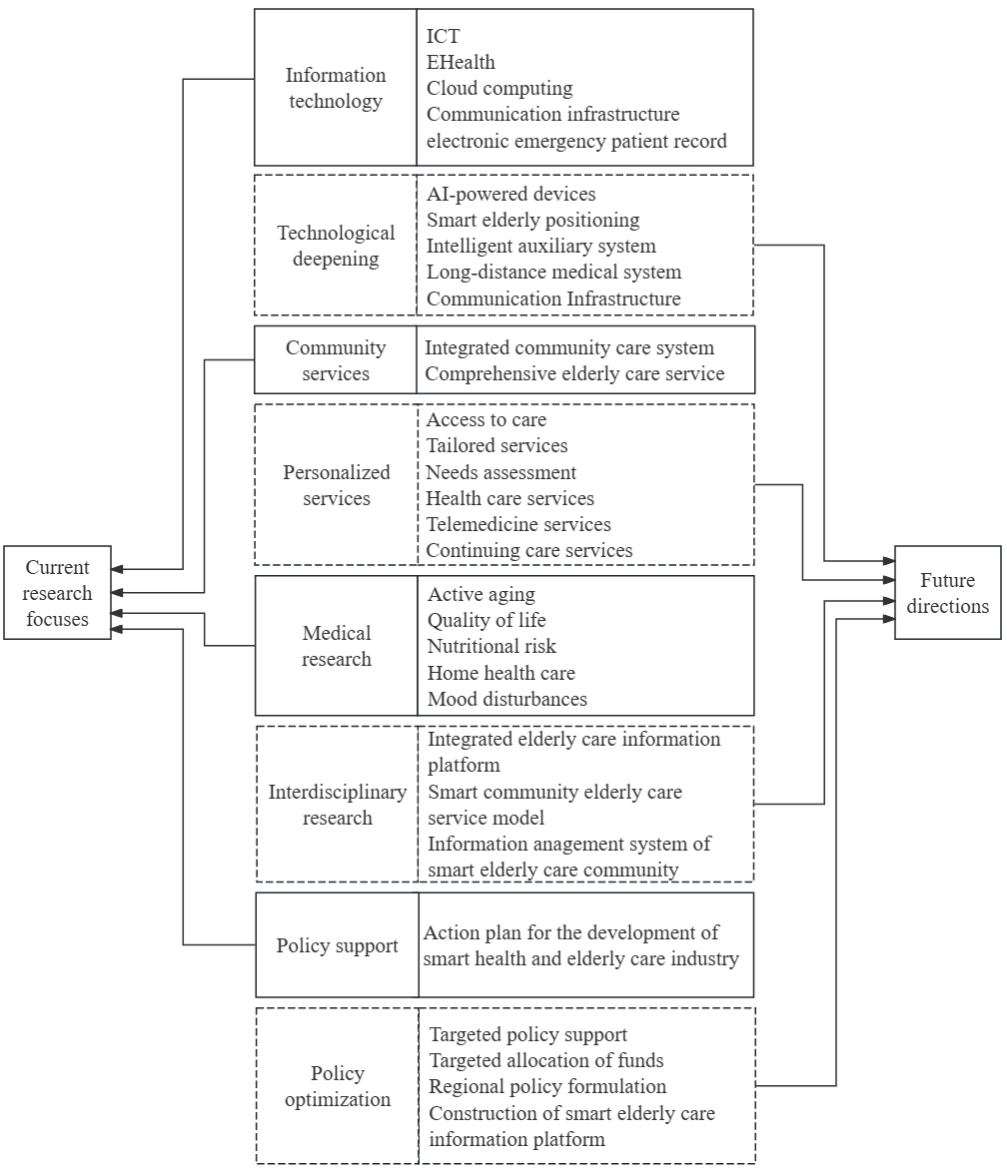
Future research directions.

This study identified the keywords and clusters through keyword analysis and cluster analysis of relevant papers in the field of elderly care community services, clarifying the research direction and research gaps in this field. At the same time, the study also combined the digital health strategy of authoritative institutions to extract the current research status and trends in this field and summarized the key contents and future research directions, as shown in Figure 13. The following text will further discuss these issues.

(1) Conduct in-depth research on smart elderly care and health technology.
  Innovations in senior care and health technology are the current research hotspots. Future research should focus on developing intelligent technologies for elderly care, with a particular emphasis on AI-driven health monitoring, age-friendly technology design, and blockchain for data security [148,149]. Big data and artificial intelligence enable the real-time monitoring and analysis of health data for older people, offering more accurate health management solutions. Furthermore, the advancement of Internet of Things technology will lead to a greater integration of smart homes and wearable devices into the daily lives of older people, significantly improving their quality of life and convenience. Nonetheless, as intelligent care for older people and health technology continue to be refined, potential challenges must be addressed. The maturity and stability of technology require enhancement, and disparities exist in the technology acceptance and usage proficiency among older adults. Furthermore, data security and privacy protection are paramount, requiring the implementation of robust data protection measures to safeguard the personal information of older people.
(2) Research on an Integrated Community-Based Elderly Care Service System.
  Integrated community care requires coordination of cross-departmental resources. Referring to relevant reports from the Australian government, the World Health Organization, and other institutions, in the future, we should focus on standardizing service workflows and digital needs assessment tools, as well as eliminating resource duplication [150,151,152]. Furthermore, this study suggests that community services should emphasize integration, enhancement, standardization, and the judicious distribution of resources. First, service content necessitates integration and optimization, encompassing medical care, rehabilitation, caregiving, and entertainment, with tailored services provided to address the distinct needs of older adults. Second, comprehensive assessments of the needs of older people can enhance service strategies, including advancements in healthcare, telemedicine, and ongoing care services. Third, the standardization and normalization of service processes should be advanced by instituting uniform service standards and procedures to enhance service efficiency and quality. Finally, the judicious allocation and distribution of service resources must be accomplished, enhancing the utilization of human, material, and informational assets to minimize expenses while increasing efficiency. Although research on integrated community-based elderly care service systems has progressively increased in recent years, their implementation has encountered numerous challenges. Integrating service resources requires enhancement, as specific resources are duplicated or squandered. Conversely, the standardization and normalization of service processes are insufficient, resulting in cumbersome and inefficient workflows. Moreover, the professional qualifications of service personnel are inconsistent, requiring enhanced skills training and practical experience to improve their competencies.
(3) Research on Interdisciplinary Collaboration and Information Sharing.
  The research of eldercare community services is progressively transitioning from a singular medical domain to a multidisciplinary comprehensive inquiry. However, interdisciplinary collaboration must still be based on WHO interoperability standards and integrate ICN’s nurse-led coordination model, which involves developing collaborative platforms, nurse-led information hubs, and breaking down information silos [148,149,152]. In the future, the innovation and implementation of interdisciplinary collaboration models will emerge as a focal point of research, encompassing the exploration of cooperative mechanisms and models across various disciplines and evaluating the efficacy of collaboration. Furthermore, developing and enhancing information-sharing platforms for older people is essential for facilitating information exchange and collaborative efforts among diverse departments and institutions. Moreover, the implementation and advancement of IT present extensive opportunities for the sector. Emerging technologies such as blockchain and artificial intelligence can facilitate the advanced development of eldercare community service systems. Despite interdisciplinary collaboration and information sharing being considered fundamental research directions in this domain, significant challenges persist. The disparities in research methodologies and epistemologies across various disciplines augment the intricacy of interdisciplinary collaboration. Conversely, information-sharing mechanisms remain underdeveloped, with information silos presenting a significant challenge, thereby requiring immediate improvement in the standardization and regulation of information exchange.
(4) Precise policies and financial support.
  Precise policies and financial support are essential for developing information-driven elderly community services. Policy-making should draw on the WHO’s policy maturity assessment tool and refer to effective policies of many governments, focusing on dynamic policy adaptation, public–private investment framework, and performance monitoring indicators [150,151,152]. Moreover, novel mechanisms and models for financing elderly services should be implemented, including the creation of dedicated funds and the integration of social capital through various approaches to provide economic assistance for advancing elderly services. Furthermore, assessing and overseeing the efficacy of policies and financial assistance is essential. Regular evaluations clarify the outcomes of policy implementation and financial assistance, enabling the identification and resolution of existing issues. An intelligent elderly information system should be developed according to local requirements to document the health and service data of senior citizens in the area, thereby enhancing the efficiency of community elderly services. Nonetheless, specific chain reactions may transpire during the ongoing optimization of policies. The formulation and implementation of policies exhibit delays and incompatibility issues, as specific policies fail to keep pace with the rapid development of information-driven elderly community services. Conversely, the mechanisms and models for financial support remain imperfect, where issues such as inefficient fund utilization and insufficient oversight are still prevalent. Moreover, inadequate emphasis on policies and financial support by certain localities and institutions may hinder the effectiveness of implementation.

## 7. Conclusions

The issue of elderly community services is a significant topic that urgently requires attention and resolution in contemporary society. It has a profound impact on the tranquility and good health of every family. It pertains to the effective allocation of social resources, the enhancement of social security systems, and the harmonious development of intergenerational relationships. This issue not only requires responding to the needs of the elderly population but also necessitates further exploration of the frailty factors within the community. Concurrently, resolving this matter necessitates the investigation of more intelligent and humanized service models to accommodate the rapidly evolving aging society. In the past few years, information technology has garnered significant attention in the research and application fields as a viable solution to this challenge. This has resulted in significant advancements in telemedicine, intelligent assistance, and health data monitoring and analysis. Therefore, conducting a thorough and quantitative literature analysis is crucial to systematically understanding the current research status and future development directions of this field.

This study employed CiteSpace and VOSViewer software to conduct a bibliometric analysis of 461 articles published between 2004 and 2024. The analysis generated knowledge maps of collaborations, keyword changes, and citation relationships, which revealed research hotspots, evolutionary trends, and core research groups in this field. The findings suggest that research on IT facilitating elderly community services has increased significantly since 2010. Environmental science, health sciences, computer science, information systems, artificial intelligence, and social sciences are among the primary disciplines associated with community service. These fields are essential for the advancement and refinement of community service research. The United Kingdom and the United States are among the most active countries in this field of research, as evidenced by their contributions to international journals. The United States, China, and Australia are the leading countries in this field of research, as indicated by the literature analysis. Secondly, this field receives robust interdisciplinary support from research institutions led by the University of Sydney, which encompasses a variety of disciplines, including health sciences, IT, and medicine. Furthermore, John P. Hirdes and George Heckman have established collaborative teams that provide high-quality research results in this field, thereby providing significant reference value for future research. Based on the above results, this study found that (1) interdisciplinary collaboration is the most influential, stemming from its ability to integrate deep domain knowledge and cutting-edge technical expertise to solve complex real-world problems. In contrast, although collaboration within a single discipline is important, it has less transformative impact on building the required integrated service model. (2) Network fragmentation persists, especially in the form of regional clusters (such as centers in the United States, China, and Europe) and internal barriers within disciplines. The formation of this situation is closely related to factors such as policy priorities of various countries, differences in medical systems, interdisciplinary communication and method integration, and barriers to data sharing.

The investigation into IT facilitating services for the elderly community underwent three developmental phases from 2004 to 2024. The initial phase (2004 to 2011) involved exploration, concentrating primarily on “access to care”, “disability”, and “prevalence”. The second phase (2012 to 2018) was characterized by continuous expansion, during which the notion of intelligent elderly care was developed. Research focal points have progressively transitioned to “intervention”, “cognitive impairment”, “home”, and “community services”. The third phase (2019 to 2024) initiated a period of swift advancement, marked by ongoing technological and business model innovations, with research concentrating on "healthcare technology," “primary healthcare”, and “technology”. The aforementioned keywords, along with their research content and trends, not only illustrate the diversity and complexity of information research in technology-enabled elderly community services but also signify the progressive refinement and adaptation in integrating IT to address community elderly care challenges across various disciplines and regions. This has led to the formation of collaborative teams that yield high-quality research outcomes in this field, providing significant reference value for future studies.

This study delineates four principal research clusters: “community care”, “access to care”, “technology”, and “older adults”. It further encapsulates and examines prospective developmental trajectories in this domain: (1) Smart elderly care and health technology will evolve towards greater personalization and intelligence; however, challenges such as technological maturity, user acceptance, and data security must be resolved. (2) The integrated community elderly care service system will advance towards a more comprehensive and multi-tiered approach. Nonetheless, additional improvement in service integration, standardization, and the professional proficiency of participants is necessary. (3) Interdisciplinary collaboration and information exchange are anticipated to enhance the advancement of comprehensive multidisciplinary research; however, mechanisms for cooperation and information-sharing platforms require enhancement. (4) Targeted policies and financial assistance will facilitate development; however, the adaptability of policies, the efficiency of fund utilization, and the focus on local institutions require further optimization.

This study enhances comprehension of the demand for elderly care services in an aging society. It advances the application of IT in this sector, offering empirical support for developing pertinent theories. The results facilitate the analysis of application trends and the potential value of IT in elderly care services, providing a scientific foundation for policy formulation and market development. This study also provides practical references for enhancing community elderly care services empowered by information technology. However, there are limitations: (1) The database coverage is limited to the Web of Science Core Collection (SCI-EXPANDED/SSCI/A&HCI) and does not include the non-indexed literature (such as regional databases and grey literature). (2) The search strategy based on keywords may miss synonym variants, and the restriction to English-only filtering may result in language bias. (3) Despite using emergence strength analysis, emerging topics with few citations (such as AI ethics) are still prone to marginalization, and manual screening is subjective, which can lead to bibliometric bias. Therefore, the current conclusions mainly reflect mainstream trends. To gain a more comprehensive understanding of the field, it is necessary to integrate multiple databases and expand semantic searches to encompass a broader range of information.

This study anticipates that the future development of elderly care community services will focus more on personalization and intelligence, refine service directions, and strengthen interdisciplinary cooperation and information sharing to enhance the precision and efficiency of health management for the elderly.

## Figures and Tables

**Table 1:** Search phrases.

Topic	Search Terms
Information	Information OR Information Technology Integration OR
	Computerization OR Digitalization OR Automation OR Electronic
	Data Processing OR Data Processing OR Information Management
	OR Information Systems Development OR Software Development
	OR Hardware Integration
Pension	Pension OR Old-age OR Endowment OR Senior OR Retirement
	OR Elderly Care OR Aged Elderly Service OR Superannuation
	OR Nursing OR Old Age
Community	Community Service OR Community-based Services OR
	Communal Services OR Community-service OR
	Community-based Service

**Table 2:** Top 10 major journals in which articles have been published.

Rank	Journals	Numbers	Country	IF(JCR2023)
1	BMJ OPEN	24	United Kingdom	2.4
2	BMC HEALTH SERVICES RESEARCH	19	United Kingdom	2.7
3	BMC GERIATRICS	17	United Kingdom	3.4
4	HEALTH & SOCIAL CARE IN THE COMMUNITY	11	United Kingdom	2.0
5	JOURNAL OF THE AMERICAN GERIATRICS SOCIETY	10	USA	4.3
6	FRONTIERS IN PUBLIC HEALTH	10	Switzerland	3.0
7	BMC PUBLIC HEALTH	9	United Kingdom	3.5
8	GERONTOLOGIST	8	USA	4.6
9	JOURNAL OF MEDICAL INTERNET RESEARCH	8	Canada	5.8
10	PLOS ONE	7	USA	2.9

**Table 3:** Top 10 countries by cooperation intensity.

Rank	Country	Numbers	Citations	Average Citations	H-Index
1	USA	129	2684	20.81	30
2	China	86	762	8.86	15
3	Australia	68	801	11.78	16
4	Canada	57	723	12.68	16
5	United Kingdom	53	1128	21.28	19
6	Netherlands	16	353	22.06	11
7	South Korea	15	148	9.87	7
8	Sweden	11	169	15.36	6
9	Belgium	9	165	18.33	6
10	Japan	9	49	5.44	4

**Table 4:** Top ten institutions by publication volume.

Rank	Organization	Numbers	Citations	Average Citations	H-Index
1	UNIVERSITY OF SYDNEY	16	216	13.50	7
2	UNIVERSITY OF CALIFORNIA SYSTEM	13	250	19.23	7
3	UNIVERSITY OF LONDON	13	485	37.31	10
4	US DEPARTMENT OF VETERANS AFFAIRS	13	156	12.00	7
5	VETERANS HEALTH ADMINISTRATION VHA	13	156	12.00	7
6	FLINDERS UNIVERSITY SOUTH AUSTRALIA	11	100	9.09	5
7	MONASH UNIVERSITY	11	61	5.55	5
8	UNIVERSITY OF TORONTO	11	84	7.64	4
9	GERIATRIC RESEARCH EDUCATION CLINICAL CENTER	10	173	17.30	7
10	MCMASTER UNIVERSITY	10	143	14.30	6

**Table 5:** Statistical table of the top 10 authors by publication volume.

Rank	Author	Numbers	Citations	Average Citations	H-Index
1	Hirdes, John	5	52	10.40	3
2	Heckman, George A.	5	50	10.00	3
3	Georgiou, Andrew	4	48	12.00	3
4	Jorgensen, Mikaela	4	48	12.00	3
5	Westbrook, Johanna	4	48	12.00	3
6	O’Donnell, Catherine	3	202	67.33	3
7	Bruce, Martha L.	3	97	32.33	3
8	Bleijenberg, Nienke	3	79	26.33	2
9	Wallin, Lars	3	74	24.67	2
10	Siette, Joyce	3	45	15.00	3

**Table 6:** Statistical table of the top 10 journals by publication volume.

Rank	Cited Journals	Citations	Country	IF(JCR2023)
1	JAM GERIATR SOC	417	USA	4.3
2	GERONTOLOGIST	231	USA	4.6
3	JAMA-J AM MED ASSOC	193	USA	63.1
4	BMC HEALTH SERV RES	172	United Kingdom	2.7
5	AGE AGEING	170	United Kingdom	6.0
6	LANCET	168	United Kingdom	98.4
7	SOC SCI MED	164	United Kingdom	4.9
8	BMC GERIATR	158	United Kingdom	3.4
9	PLOS ONE	157	USA	2.9
10	BMJ-BRIT MED J	145	United Kingdom	93.6

**Table 7:** Top 10 cited authors.

Rank	Cited Authors	Total Link Strength	Citations
1	BRAUN, V	44	34
2	HIRDES, JP	141	30
3	MORRIS, JN	142	28
4	FOLSTEIN, MF	47	25
5	LAWTON, MP	30	22
6	PATTON, MQ	35	20
7	CRESWELL, JW	34	18
8	WARE, JE	30	18
9	COLEMAN, EA	39	16
10	VENKATESH, V	33	16

**Table 8:** Top 10 most cited documents.

Rank	Title	Journal	Author(s)	Citations
1	A new framework for developing and evaluating complex interventions: update of Medical Research Council guidance [118]	BMJ-BRITISH MEDICAL JOURNAL	Skivington K	4
2	Global estimates of the need for rehabilitation based on the Global Burden of Disease study 2019: a systematic analysis for the Global Burden of Disease Study 2019 [119]	LANCET	Cieza A	4
3	Frailty Consensus: A Call to Action [120]	JOURNAL OF THE AMERICAN MEDICAL DIRECTORS ASSOCIATION	Morley JE	3
4	Reducing suicidal ideation and depressive symptoms in depressed older primary care patients—A randomized controlled trial [121]	JAMA-JOURNAL OF THE AMERICAN MEDICAL ASSOCIATION	Bruce ML	2
5	Integrating telecare for chronic disease management in the community: What needs to be done? [122]	BMC HEALTH SERVICES RESEARCH	May CR	2
6	Navigation roles support chronically ill older adults through healthcare transitions: a systematic review of the literature [123]	HEALTH & SOCIAL CARE IN THE COMMUNITY	Manderson B	2
7	Storage symptoms of the bladder: prevalence, incidence and need for services in the UK [124]	BJU INTERNATIONAL	McGrother CW	2
8	What prevents older people from seeking treatment for urinary incontinence? A qualitative exploration of barriers to the use of community continence services [125]	FAMILY PRACTICE	Horrocks S	2
9	Aging in Community Developing a More Holistic Approach to Enhance Older Adults’ Well-Being [126]	RESEARCH IN GERONTOLOGICAL NURSING	Davitt JK	2
10	Evaluation of outcomes with citalopram for depression using measurement-based care in STAR*D: Implications for clinical practice [127]	AMERICAN JOURNAL OF PSYCHIATRY	Trivedi MH	2

## Data Availability

The original contributions presented in this study are included in the article. Further inquiries can be directed to the corresponding author.
